# Shading and Water Addition Alleviate the Elemental Limitations of the Early Restoration Community in a Stressful Environment

**DOI:** 10.3390/plants13182626

**Published:** 2024-09-20

**Authors:** Fajun Chen, Gaojuan Zhao, Youxin Shen, Hong Zhu, Zhenjiang Li, Beilin Tan

**Affiliations:** 1 CAS Key Laboratory of Tropical Forest Ecology, Xishuangbanna Tropical Botanical Garden, Chinese Academy of Sciences, Menglun 666303, China; chenfajun@xtbg.ac.cn (F.C.); zhaogaojuan@xtbg.ac.cn (G.Z.); zhuhong@xtbg.ac.cn (H.Z.); lizhenjiang@xtbg.ac.cn (Z.L.); tanbeiling@163.com (B.T.); 2 University of Chinese Academy of Sciences, Beijing 100049, China; 3 College of Life Science, Neijiang Normal University, Neijiang 641100, China

**Keywords:** ecological stoichiometry, nutrient limitation, forest restoration, topsoil translocation, leaf functional traits

## Abstract

Shading and water addition are essential management measures to improve seed germination and early seedling survival; however, little is known about their effects on leaf stoichiometry and nutrient status. We established 90 plant communities with shading and water addition gradients on a rocky hill; leaves of their dominant woody plant species were collected to measure elemental concentrations, and then, stoichiometric variation and nutrient status were analysed. The results showed that the overall effects of shading and water addition significantly altered the concentrations and ratios of nutrient elements; shading largely affected leaf K and P, while water addition mainly affected leaf N and P. The interactions between shading and water addition were significant for most species but disappeared at the community level. Consequently, the nutrient status in leaves was improved by promoting the concentrations and balances of nutrient elements. However, the responses to shading and water addition were marked by species-specific differences, with some plants forming a sensitive group and others distinguished by conservatism. Our findings show that management of the physical environment could improve nutrient element utilization in leaves and alleviate the nutrient limitations. For our site conditions, mild shading (25–35%) and adequate water addition (30 L·m^−2^) in the early stage of vegetation restoration is recommended to advance community assembly by improving nutrient physiology, directly diminishing the stress of water scarcity and excessive irradiation. These findings explore the underlying mechanisms of shading and water addition that could promote community development and provide guidance for restoration practice.

## 1. Introduction

Habitat loss is one of the most pressing threats to biodiversity in regional and global ecosystems [1]. Natural forests of various types are garnering increasing attention for their high biodiversity and role in mitigating climate change, as well as other ecosystem functions and services [2]. As large areas of natural forests are being damaged by human activity and global change, such as urban expansion and conversion to agricultural land, the quality and function of forests are also threatened by multiple interacting factors [2,3]. This environmental problem is much worse when rocky desertification is superimposed upon arid and semiarid areas [4]. Accordingly, vegetation restoration is imperative to establish forest communities for filling gaps in fragmented habitats and improving the functioning of degraded forests. For ecological restoration, obtaining diverse species of seedlings and enough individuals of them is essential for robust community establishment [5,6]. Moderate microclimatic conditions, such as suitable light and water resources, are crucial for supporting the early assembly of a new plant community [6,7,8]. This is especially pertinent given the harsh conditions of most degraded lands and the fragility of seedlings; hence, habitat-modifying interventions are necessary to maintain and accelerate the construction of a plant community. 

Rainfall is the main form of water supplemented to most terrestrial ecosystems, whose regimes at various scales are shifting due to climate change [9], such that water availability is fluctuating more extremely, and drought stress could become a common event in certain areas [4,10]. Light heterogeneity is extremely high in forest communities from the canopy down to the understory and ground surface. Although competition for light is common and critical to plant species’ dynamics [11,12], the seedlings of most tree species often need some degree of shading to avoid stress generated by excessive light levels, being more likely to survive and grow better with shade facilitation [7,13,14], thereby driving community assembly and dynamics [12]. Thus, environmental interventions, such as applying shading and adding water, are usually conducted to foster seedlings and to study the impacts of habitat changes [6,15]. Previous studies have reported significant effects of light and water on the morphological and physiological traits of plants and their survival and growth, whereas interactive effects between light and water remain contentious [13,16,17,18]. Thus, multiple species and continuous gradients of key abiotic factors are needed to further explore such interaction effects [16]. For newly restored communities, the responses of co-occurring species with different traits to similar light and water changes are also not well explored. 

Besides light and water, elemental concentrations and their ratios in leaves are tightly correlated with the performance of individual plants and communities [19,20], constituting important resources that can affect the structure and dynamics of restored vegetation [21,22]. Nitrogen (N) and phosphorus (P) are widely used in studies on elemental utilization and functional traits because they are the most common limiting elements for plants and ecosystems [23]. Meanwhile, potassium (K) is drawing more attention for its role in maintaining the physiological balance and growth of plants [24,25]. Although stoichiometric indices are often viewed as homeostatic, changed stoichiometric characteristics of plant species or communities could reflect adaption to environmental variation [20,26]. Leaf stoichiometry is considered a reliable indicator of a plant’s nutrient status [23,27,28]. The analysis of leaf stoichiometry patterns is widely used to explore the mechanisms underlying biogeographic variation [10,29,30], climate responses [20,31], and other ecological processes [19,20,32]. Thus, leaf nutrient status may also be altered by light and water conditions, and this could theoretically further influence the restoration process. Yet, so far, because it is difficult to manipulate a natural forest and obtain a relatively homogeneous background condition, how shading and water addition affect the stoichiometric characteristics of natural communities remains poorly understood in field management practices. 

Here, we established restoration communities via topsoil replacement whose plant individuals were acquired from a soil seed bank and were similar to local natural forest vegetation [6]. We quantitatively investigated the impacts of shading and watering on the leaf stoichiometry of the main woody plants in these early-stage restoration communities. Their nutrient status and patterns of change were evaluated, with a view towards deriving optimal management. We tested three hypotheses: (a) At both the community and species level, light and water interventions could promote plants’ nutrient utilization and shift their degree of limitation by N or P. (b) Co-occurring plants should harbour species-specific nutrient adaptation strategies along the light and water gradients. If so, some sensitive species and elemental indices will be more suitable as indicators of plant–environment interactions and the restoration process. (c) In terms of the plant community’s nutrient status, an optimum combination of shading and watering can be found to foster early-stage vegetation restoration.

## 2. Results

### 2.1. Concentrations and Ratios of Nutrient Elements in the Communities

For plant species in the restoration communities, their leaf traits differed in terms of their nutrient elemental concentrations and ratios (Table S1). The ranges were 13.75 to 34.34 g·kg^−1^ for N, 0.50 to 2.07 g·kg^−1^ for P, and 2.89 to 23.92 g·kg^−1^ for K. For the ratios, their values ranged widely, from 8.87 to 44.63 for N:P, 0.68 to 7.67 for N:K, and 0.038 to 0.24 for P:K. N, K, and their relative ratios exhibited high variation (variation coefficients > 20%). All of the concentrations and stoichiometric ratios were significantly different among the 11 woody species (*p* < 0.001) and showed obvious species-specific features. The mean concentrations of total elements in plot soils were 3.91 g·kg^−1^ for N, 0.66 g·kg^−1^ for P, and 4.35 g·kg^−1^ for K; N was similar to the soil in the natural forest, but P and K were lower (*p* < 0.01). 

At the community level, the total stoichiometry of average leaf C, N, P, and K was 465.57: 20.40: 1: 10.69; when weighted by the leaf biomass of species, the values of the four elements were 463.88: 20.76: 1: 11.99. The concentration of P in the weighted community was higher than that directly calculated by the species mean.

**Figure 1:**
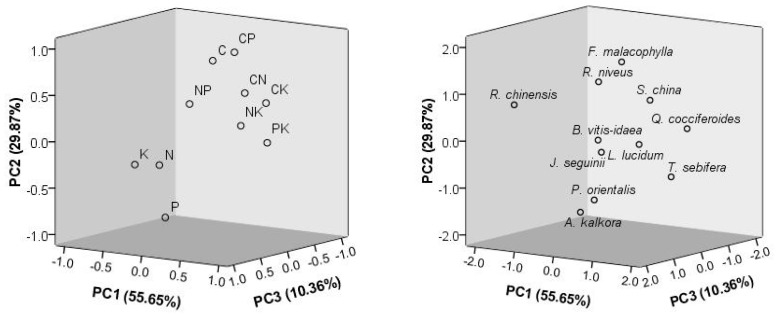
Stoichiometric relationships of leaf elemental concentrations and ratios (**left**) and of the 11 studied woody plant species (**right**). PC1-3 represent the first three principal components obtained by PCA, and the numbers in brackets are the proportion of variation explained. CN, CP, CK, NP, NK, and PK represent the ratios of the two elements.

In total, 95% of the variation was explained by the first three principal components (Figure 1). The concentration of K had high negative loadings on the first PCA axis, while its ratios with other elements had positive loadings. Traits associated with P (including relative ratios) had high loadings on the second PCA axis, and traits associated with N had high loadings on the third PCA axis. The scores of plant species in three axes are also shown in Figure 1, where evidently the distribution of the 11 species is sporadic.

### 2.2. Effects of Shading and Water Addition on Stoichiometric Traits in the Communities

Multiple linear regressions were used to test the effects of shading and watering on the leaf element concentrations and ratios at the community level (Table 1). When using the arithmetical mean, all regression models were significant at 0.05, except for C, C:N, and N:K. Shading changed the leaf P, K, C:P, C:K, N:P, and N:K ratios (*p* < 0.05). Water addition increased the leaf concentrations of N and P and changed the ratios of C:P, N:P, and P:K (*p* < 0.05). The community-weighted mean (by abundance and leaf mass) showed similar patterns, except for the effects of watering on N and P. Shading significantly affected the leaf concentrations of P and K, and shading also remarkably changed the C:P, C:K, N:P, and N:K ratios (*p* < 0.05). Meanwhile, an effect of water addition upon P was detected in the abundance-weighted analysis, while an effect on N was observed in the leaf-mass-weighted analysis. No significant interaction effect was observed between shading and water addition except for C and C:N. 

**Figure 2:**
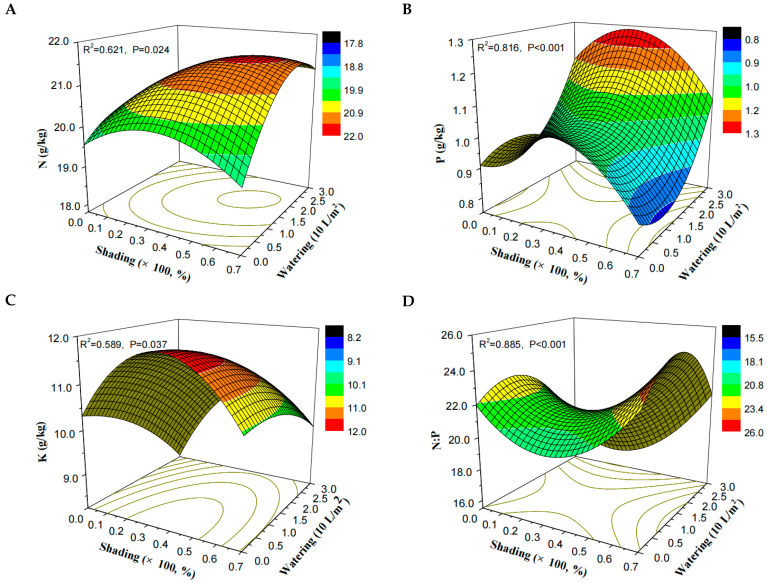
Responses of N, P, and K concentrations (**A**–**C**) and N:P (**D**) of nutrient elements in leaves to shading and water addition. The surfaces were generated according to the fitted models (binary quadratic functions). Values along the axes of shading and watering were rescaled to a similar order of magnitude and to obtain the coefficients of the fitting equations.

Overall, the shading treatment affected more stoichiometric traits than water addition in this early-stage plant restoration community, and the effects of shading on P, K, and the relative ratios were stable. The influences of watering on leaf P, C:P, and P:K disappeared in leaf-mass-weighted community analysis, whereas its influence on leaf C and C:N was discernible. According to the binary quadratic function, higher values of N, P, and K and a lower value of N:P were observed in response to mild shading combined with maximum water addition (Figure 2). The N:P ratio was a sensitive index; it responded significantly to the changed levels along both shading and watering gradients in three analytical methods (f ≥ 12.929, *p* < 0.001).

### 2.3. Species-Specific Variation and Comparisons with the Control and Natural Forest

**Figure 3:**
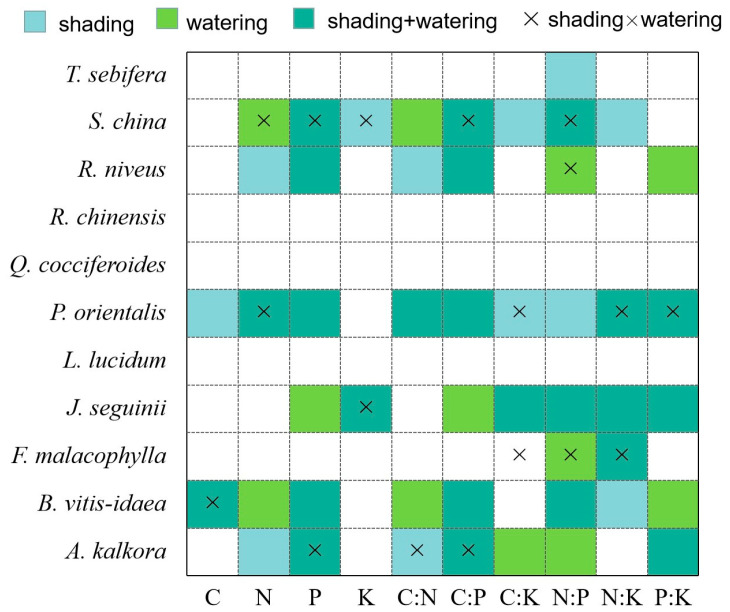
Effects of shading and water addition on the stoichiometric characteristics in leaves of 11 plant species. Regression analysis was conducted to test the significance of the models and coefficients. Coloured cells had significant effects at the 0.05 alpha level; light blue and bright green denote the effects of shading and water addition, respectively; greenish blue indicates that both the effects of shading and water addition were observed. Interactive effects of shading and water addition are shown by the “×” in the cells.

The plant species responded differently to the shading and watering treatments. As Figure 3 shows, eight of eleven species underwent significant changes in response to habitat management (including *Sapium sebiferum* with only variation in N:P), whereas three species responded negligibly to both treatments. Further, the leaf K concentration was the index for which the fewest species were affected by the treatments, while the N:P ratio was changed for most of them. Interactions between shading and water addition effects were common at the species level, especially in *Smilax china* and *Paliurus orientalis*. 

When treatments with shading and/or watering were compared to the control quadrats, greater N, P, and K concentrations were detected in certain species (Figure S1). Four, five, and two species had significant increases in N, P, and K, respectively (t ≥ 3.793, *p* < 0.01), but N decreased in *S*. *china*, and K decreased in *Albizia kalkora*. When compared with the natural forest, five species in the restoration community had a lower P concentration (t ≤ −3.263, *p* < 0.05), and four species had a lower K concentration (t ≤ −7.590, *p* < 0.01), while the other species had similar concentrations between the two systems. Most elemental ratios were significantly altered in the managed habitats (Figure S1). For seven of the eleven species, their N:P ratios were lower in managed habitats than in the control (t = 8.893 or t ≤ −2.733, *p* < 0.05); these indexes were higher than in the natural forest in six of nine species (*p* < 0.05). More species had changed ratios with respect to N:P than N:K and P:K. In total, the status of leaf nutrients with habitat management was a condition between the control and the natural forest.

## 3. Discussion

This study was conducted in plant restoration communities established by soil translocation to which shading and water addition treatments were applied in the field. Our findings demonstrated that shading and water addition could differentially alter stoichiometric characteristics and then improve the nutrition status of restored vegetation in the absence of any nutrient addition. Further, this study highlights the different responses of co-occurring species, as well as contrasting species and community level responses to habitat management. 

### 3.1. Elemental Characteristics and Their Responses to Shading and Water Addition 

At the community level, the N concentration we found was higher than the mean of plants in China (14.14 g·kg^−1^) and the global mean (18.9 g·kg^−1^), while the P concentration was lower than the national mean (1.11 g·kg^−1^) and global mean (1.2 g·kg^−1^) [19,33]. In karst areas, the N concentration was similar, and P was lower than the reported concentrations (20.62 and 1.45 g·kg^−1^, respectively) for plants from southwestern China [34]. In our experiment, the overall N:P was >20.0, which is higher than the national level (mean = 14.4) [35], and this value remained high even when we excluded the legume tree species. In addition, the total P of soils in our plot (0.66) was lower than in most areas of China and other ecosystems in the same region [22,36]. Hence, according to the standards of nutrient limitation [23,27], the restoration community showed P limitation.

Leaf nutrient traits are sensitive to local physical environment changes, though they respond differently. At the community level, all concentrations and ratios were affected by shading and water addition to some extent, of which N:P was the most sensitive and reliable. These results are consistent with some previous studies (e.g., [11]). There are three main reasons for the changes. First, shading could directly reduce excessive radiation to protect the seedlings, and this moderated habitat would benefit their nutrient utilization due to less stress incurred [13,15]. Moreover, water not only changes the conformation of elements and accelerates their movement in soil but also facilitates their absorption by roots [37,38]. In addition, shading and watering could change the physical conditions of the habitat, whose moderate state could improve the growth of individuals [8,13,15]. Thus, as a plant’s demand for elements increases due to active metabolism [37], a greater content accumulation in its leaves would ensue. As key nutrient elements, N, P, and K in leaves are related to many plant physiological process; hence, their dynamics with environmental change are frequently observed [20,28].

However, we found divergent effects of shading and water addition on elemental characteristics. The shading mainly affected the leaf P and K concentrations, while water addition mainly affected the leaf N and P concentrations at the community level. Leaf N and P are usually coupled in ecological process; their concentrations were reduced by higher light and drought intensification [11,13,39]. Yet, asymmetrical effects were also observed in some studies: leaf P (not N) was mainly affected by soil water in desert shrubs [40], while leaf N was strongly changed by more rainfall, and along drought gradients [10,41], they are controlled by different mechanisms [42]. Our study’s findings are in contrast with a recent report that revealed that water addition did not alter non-metallic elements in plants [31]. Interestingly, shading seemed to play a more important role than watering in controlling K variation in our study, although K is strongly related to the plants’ tolerance of water stress [20,24]. Besides preventing the harm caused by excessive light, shading can alter the microclimate and change leaf temperatures and water potential [43,44]; thus, it may relieve drought-induced stress. However, our findings showed that the K concentration was higher in moderate shading and lower in too slight or severe shading. These results suggest that the effects of shading on leaf K mainly arise from a plant’s direct demand; an appropriate shade environment could promote plant metabolism and growth. An increase in leaf K could be explained by its various metabolic and physiological functions and relationship to plant growth processes [24,25]. Meanwhile, the effects of water addition might be limited to K dynamics of leaves in dry areas and seasons because of the speedy infiltration of water. Root depth is also a factor which influences how the K contents of plant species are modulated [31]. Concerning N, it is correlated with photosynthesis and growth [23,45], and the condition of soil water can shift its N availability [38]. Thus, the water addition had a significant effect on the N in leaves in our experiment. When using the weighted community values, the responses of leaf N and P to water addition showed differing performances, which could be chiefly explained by distinctive contributions of biomass of various species.

### 3.2. Interactive Effects of Shading and Water Addition on Leaf Stoichiometry

This study revealed that the effects of shading and watering upon leaf stoichiometry were not always consistent at the community and species level, especially for interactions between shading and watering. The interactive effects of light and water on plant performance remain controversial, with some studies supporting the independent functions of light and water and others claiming they exert interactive effects [13,16,17]. Shade can affect the microclimate, including humidity [43]; thus, light and water conditions may be related. In our study, we found significant interactions in most species, but such patterns vanished at the community level. When considering species separately, the response of a single species, which reflects a specific adaption strategy, can be analysed. When considering a plant community, the specific responses of diverse species might be mixed, such that the overall effects of shading and water might now show different patterns. Plant species in our restoration community had distinctive stoichiometric responses, and they could be divided into two types of groups, sensitive and conservative. Most of the 11 species changed their concentrations and ratios of nutrients in leaves in response to habitat management, evincing obvious plasticity in their leaf nutrient elements; however, other species, such as *Ligustrum lucidum* and *Quercus cocciferoides*, displayed only slight changes, implicating relatively conservative responses to shading and water addition. These contrasts indicate that different nutrient-enhancing pathways exist in plants through which they respond to and match the environmental variation generated by shading and watering [10,11,16,46].

### 3.3. Limitation of Nutrient Elements and Implications for Vegetation Restoration

Plants tend to suffer nutrient limitation because of relatively low elemental concentrations and finite soil resources [22,36]. This limitation was directly reflected in the infertile soil and plant leaves with low P concentration and high N:P in this study. After shading and watering the communities, the characteristics of elemental concentrations and ratios in treated conditions were between the status of an unmanaged community and the natural forest; some proper combinations of shading and water addition were similar to the natural forest. Leaf N and P concentrations and their balance were all improved. These findings show that additive benefits of alleviating N and P limitation to plants could be generated by shading and watering, increasing the number of species and individuals in the restoration community [6]. Considering that P and K were relatively low in the soil and that there was a significant P limitation for the plants, obviously promoted balances of N:P, N:K, and P:K in most species should benefit their seedlings’ growths. Alterations in nutrient limitation are also observed by precipitation and light in other ecosystems; the variation in ratios may arise from changing elemental concentrations which are disproportionally increased or decreased [29,47,48].

Considering the positive effects on nutrient status, proper habitat-modifying treatments that facilitate plant species’ performance could be selected in restoration projects. As shown in Table 1 and Figure 2, shading mainly impacted leaf P and K and showed a unimodal pattern with a maximum under slight shading. Arguably, then, shading should be implemented in patches earmarked for restoration because it is a cheap and convenient method. Water addition increased both leaf N and P concentrations and can also change N uptake rates and forms [49], but obtaining water may be difficult in some restoration locations; where possible, watering treatments are thus suggested, by using a proper volume in the early stage of restoration in arid areas or seasons. In prior studies, the results demonstrated that shading and water addition can promote species diversity, seedling abundance, and individual performance [6,50], further emphasizing the necessity of light and water interventions. In our study, mild shading (about 25–35%) and adequate water addition (30 L per m^2^) in the early stage of establishing communities are suggested to promote the plants’ nutrient status and the community assembly process.

## 4. Materials and Methods

### 4.1. Study Site

This research was conducted in Jianshui County in the Yunnan Province, Southwest China. This area is identified as a typical karst region with a hot and seasonal dry climate [4,6]. Here, the mean annual air temperature is 18.5 °C, and the mean annual precipitation amounts to 850 mm; most of it falls in the rainy season (May to September) [6]. Photosynthetically active radiation (PAR) is above 2000 μmol·m^−2^·s^−1^ in the summer on a clear midday. The soil in the study area is red earth; the concentrations of N, P, and K of the topsoil (20 cm) are 3.91 g·kg^−1^, 0.66 g·kg^−1^, and 4.35 g·kg^−1^, respectively. The natural vegetation is sclerophyllous evergreen broad-leaved forest, where the main plant species in the forest include *Quercus cocciferoides*, *Fraxinus malacophylla*, *Smilax china*, etc. The field experiment plot (23°41′ N, 102°56′ E, at 1500 m a.s.l.) was established in 2017 on a karst hill with rocky desertification; the vegetation is degraded grassland with sparse dwarf shrubs.

### 4.2. Experimental Design

**Figure 4:**
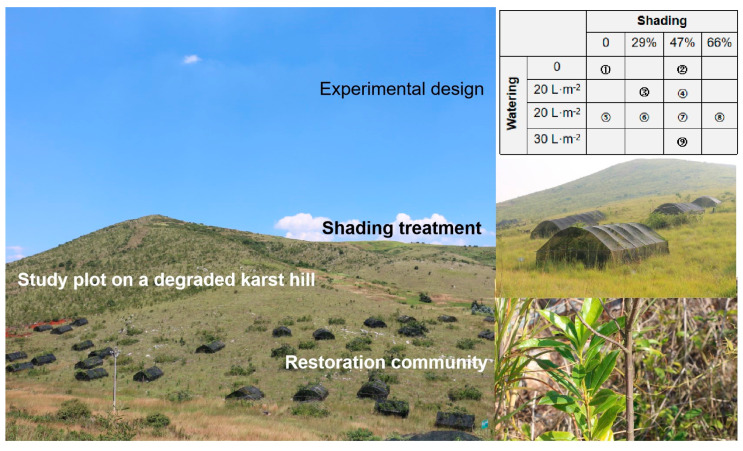
The study area and experiment of forest restoration on a karst hill in southwestern China. Ninety quadrats (5 × 5 m) were established for experimental treatments. Vegetation and topsoil in all the quadrats were removed before placing the topsoil from a local forest; then, shading and water addition were applied, and nine environmental conditions (⓵–⓽, upper right) were generated.

Ninety quadrats (each 5 × 5 m) with different shade and water addition were set up in five blocks in the restoration plot (Figure 4). To obtain the soil seed bank and minimize edaphic heterogeneity among plots, the surface-layer soil (0–10 cm depth) was replaced with the topsoil of a local natural forest, located 5 km from the study site. In each block, 18 quadrats were randomly arranged with a buffer of at least 3 m space between adjacent quadrats, to which different shading and watering management treatments were applied. Black shade nets were carefully selected to generate light conditions that spanned four levels—*mild*, reducing 29% of the total solar radiation under open circumstances; *moderate*, reducing 47% of the total; *severe*, reducing 66% of the total; *open*, 100% of natural light (the control). These light conditions of photosynthetically active radiation were determined by SKS-1110 (Skye Instruments Ltd., Powys, UK). Water addition was executed every month and consisted of four levels: *no addition* as the control, *low* (10 L·m^−2^), *intermediate* (20 L·m^−2^), and *high* (30 L·m^−2^), generated by manually watering quadrats during the first 15 months. Consequently, nine combinations with ten replicates (natural light with no addition and intermediate watering, mild shading with low and intermediate watering, moderate shading with all four levels of watering, and severe shading with intermediate watering) were set according to the quadratic saturation D-optimum design [6].

### 4.3. Sampling and Measurement

In August (i.e., peak of vegetation growth) of 2019, healthy fully expanded leaves were collected from all quadrats. Eleven common woody species (seven trees, three shrubs, and one liana) accounted for over 85% of the total coverage and represented nine families, being the focus of this research (Table 2). Three individuals (if sufficient) per species were randomly selected, from which 6–20 leaves per plant were collected within every quadrat. The number of sampled leaves depended on the relative leaf sizes of different species. The leaves of each species from five quadrats (randomly selected one in each block) of each treatment were pooled to obtain a sample; then, leaves from other quadrats were used as another replicate sample. Leaf samples of the same species from multiple individuals were also collected in a nearby natural forest for further comparison. Furthermore, surface soils (0–20 cm) from five sites in the study plot and the natural forest were sampled to analyse the main nutrient elements [22].

All leaf samples were cleaned and oven-dried at 65 °C for 48 h and then ground into a fine powder for their chemical analyses. Total C and N concentrations were quantified by an elemental analyser, Vario MAX CN (Elementar Ltd., Frankfurt, Germany). To measure P and K, 0.3 ± 0.02 g of each leaf sample was digested with 10 mL HNO_3_ and 2 mL HClO_4_ in a digestion apparatus, and then, HCl and ultrapure water were added sequentially to obtain a solution; then, the total P and K concentrations were determined by an inductively coupled plasma atomic emission spectrometer iCPA6300 (Thermo Fisher Scientific, Waltham, MA, USA) [51]. The elemental ratios were calculated with the concentrations on a dry mass basis.

### 4.4. Statistical Analysis

To evaluate the effects of environmental management at the community level, the community-weighted means of leaf nutrients were calculated based on the species’ abundance and leaf biomass because the study species have different quantities and sizes of individuals. Firstly, the number of individuals per species was also counted, avoiding plants which were too small with few leaves. Subsequently, the leaves per plant were also counted, with the number of individuals sampled per species proportional to the relative species abundances. The mean weight of leaf dry material was obtained after drying the samples’ leaves for their elemental measurement. Thus, each stoichiometric index could be calculated at the community level in three ways (see the details in Table S2): (a) simply averaging values of the 11 species; (b) calculating the mean of 11 species weighted by the abundance of their individuals, with the ratios of N:P, etc., calculated by the mass of C, N, P, and K; and (c) calculating the mean of 11 species weighted by the dry biomass of their leaves.

All the statistical analyses were implemented in SPSS 22.0 and Origin 7.0 software. The principal component analysis was used to analyse the characteristics of elements and species. We used multiple linear regression (fitting a binary quadratic function) to test the quantitative effects of shading and water addition on the element concentrations and ratios of leaves in the plant restoration communities [6]. To compare the performance of the same species under the shading and/or watering treatments to the control and natural forest, all treatments with shading and/or watering were pooled as a group; then, a one-sample *t*-test was used to determine the difference from control treatments and natural forest. The differences in soil nutrients between the study plot and natural forest were determined by an independent samples *t*-test.

## 5. Conclusions

Our study provided insight into the mechanistic effects of shading and water supply upon plant species and communities based on their nutrient status, and species-specific and scale-dependent responses existed in the restoration community. For most species, N:P is a reliable index for predicting a changed nutrient status along environmental gradients. Habitat management can improve the nutritional performance of plants by enhancing their nutrient acquisition and balance without adding fertilizer. Our intervention alleviated P limitation in the early community; therefore, such manual interventions are necessary in restoration areas (considering other benefits), especially where natural succession is impeded or rapidly restored vegetation is needed. According to our study, mild shading (varying with restoration locations) coupled with a relatively adequate water addition are recommended to alleviate stressful effects in practice.

## Figures and Tables

**Table 1:** The effects of shading and water addition treatments on the elemental concentrations and ratios of leaves at the community level. The arithmetic mean is the average value of 11 plant species for a given index; the mean weighted by abundance is calculated by measured values and the numbers of individuals per species, and the mean weighted by leaf biomass is calculated by measured values, individual numbers, and the leaf biomass of species.

	T. C (g·kg^−1^)	T. N (g·kg^−1^)	T. P (g·kg^−1^)	T. K (g·kg^−1^)	C:N	C:P	C:K	N:P	N:K	P:K
Community mean, arithmetic										
Sha × sha	2.115 **	−0.883	−1.649 **	−1.554 *	1.007	2.214 **	1.580*	1.836 **	0.984	−0.268
Shading	−1.873 *	0.935	1.703 **	1.827 **	−0.939	−2.273 **	−1.924 **	−1.869 **	−1.375 *	0.080
Wat × wat	0.864	−1.317 *	1.472 **	−0.426	1.568	−1.200 **	0.886	−2.077 **	0.924	1.942 **
Watering	−0.324	1.114	−0.818	−0.223	−1.310	0.726	−0.450	1.396 **	−0.393	−1.034 *
Sha × wat	−0.582	0.712	−0.065	0.314	−0.563	0.065	−0.107	0.405	0.104	−0.216
F	2.414	3.932	10.617	3.439	1.887	11.542	3.628	18.431	3.074	10.306
*p*	0.098	0.024	0.000	0.037	0.171	0.000	0.031	0.000	0.051	0.001
Community mean, weighted by abundance										
Sha × sha	0.409	−0.538	−1.373 **	−1.263 *	0.264	1.768 **	1.294 *	1.720 **	0.875	0.039
Shading	0.976	−0.670	1.223 *	1.996 **	1.093	−1.405 *	−1.924 **	−2.133 **	−1.758 *	−0.511
Wat × wat	0.769	−0.439	1.811 **	0.037	0.690	−1.550 *	0.326	−2.072 **	0.491	1.472 **
Watering	0.085	0.235	−1.141 *	−0.339	−0.320	1.098 *	0.034	1.338 **	−0.169	−0.701
Sha × wat	−1.338 *	1.085	−0.06	−0.142	−1.128 *	−0.050	0.046	0.622	0.267	−0.084
F	3.462	2.872	8.911	5.961	4.508	6.400	4.688	15.390	9.144	6.425
*p*	0.036	0.062	0.001	0.005	0.015	0.004	0.013	0.000	0.001	0.004
Community mean, weighted by leaf biomass										
Sha × sha	−0.016	0.091	−1.602 *	−0.976	−0.055	1.696 *	1.076	1.954 **	1.007 *	0.013
Shading	1.189 *	−0.727	1.379 *	1.224 *	0.988	−1.260 *	−1.236 *	−2.179 **	−1.451 **	−0.382
Wat × wat	1.203 *	−1.227	0.745	−0.409	1.495 *	−0.451	0.479	−1.580 **	−0.152	0.786
Watering	−0.174	1.652 *	−0.059	0.162	−1.564 **	−0.101	−0.054	1.072 *	0.619	−0.013
Sha × wat	−1.007 *	0.091	0.000	0.665	−0.463	−0.125	−0.822	0.165	−0.624	−0.700
F	9.853	3.852	5.426	4.297	5.203	4.345	4.047	12.929	8.816	3.913
*p*	0.001	0.026	0.008	0.018	0.009	0.017	0.022	0.000	0.001	0.025

Note: A regression model for a binary quadratic function was applied to test the explanatory ability of the experimental factors. Values within the same row are standardized coefficients for the factors in the fitted equations; values within columns are the corresponding F values for the fitted ANOVAs for each response variable. The bold numbers mean variables with statistical significance, * represents significance at 0.05, and ** represents significance at 0.01. “sha” is the abbreviation of shading, and “wat” is the abbreviation of watering.

**Table 2:** The checklist and basic information of plant species in this study.

No.	Species	Family	Genus	Growth Form	Leaf Habit
1	*Albizia kalkora* (Roxb.) Prain	Fabaceae	*Albizia*	Tree	Deciduous
2	*Breynia vitis-idaea* (Burm. F.) C. E. C. Fischer	Phyllanthaceae	*Breynia*	Shrub	Deciduous
3	*Fraxinus malacophylla* Hemsl.	Oleaceae	*Fraxinus*	Tree	Deciduous
4	*Jasminum seguinii* Lévl.	Oleaceae	*Jasminum*	Shrub	Evergreen
5	*Ligustrum lucidum* Ait.	Oleaceae	*Ligustrum*	Tree	Evergreen
6	*Paliurus orientalis* (Franch.) Hemsl.	Rhamnaceae	*Paliurus*	Tree	Deciduous
7	*Quercus cocciferoides* Hand.-Mazz.	Fagaceae	*Quercus*	Tree	Semievergreen
8	*Rhus chinensis* Mill.	Anacardiaceae	*Rhus*	Tree	Deciduous
9	*Rubus niveus* Thunb.	Rosaceae	*Rubus*	Shrub	Deciduous
10	*Smilax china* L.	Smilacaceae	*Smilax*	Liana	Evergreen
11	*Triadica sebifera* (Linnaeus) Small	Euphorbiaceae	*Triadica*	Tree	Deciduous

## Data Availability

Data are available in a publicly accessible repository.

## Supplementary Materials

The following supporting information can be downloaded at https://www.mdpi.com/article/10.3390/plants13182626/s1. Table S1: The element concentrations and ratios in leaves in the restoration community; Table S2: Stoichiometric indices of leaves were calculated at the community level in three ways; Figure S1: Comparison of elemental concentrations and ratios of the same species growing in managed treatments with the counterparts in the control treatment and a natural reference forest.
