# Supplementary material for: Shading and Water Addition Alleviate the Elemental Limitations of the Early Restoration Community in a Stressful Environment
Source: Plants (Basel). 2024 Sep 20;13(18):2626. doi: 10.3390/plants13182626 (PMC11434709; doi:10.3390/plants13182626)
Supplement: Supplementary file 1 [file plants-13-02626-s001.zip › plants-3202932-supplementary.pdf]

Supplementary Materials

Table S1 The element concentrations and ratios in leaves in the restoration community. The maximum and minimum values were selected in the concentrations or ratios of certain species in all treatments, and the coefficients of variation were calculated by the mean of 11 plant species.

	T. C(g/kg)	T. N(g/kg)	T. P(g/kg)	T. K(g/kg)	C:N	C:P	C:K	N:P	N:K	P:K
Mean	473.77	20.76	1.02	10.85	23.90	491.18	53.92	21.15	2.39	0.11
Maximum	509	34.34	2.07	23.92	34.76	996.10	170.62	45.71	8.41	0.26
Minimum	424	13.75	0.50	2.89	14.56	218.12	18.83	8.87	0.66	0.04
Range	85	20.59	1.57	21.03	20.2	777.98	151.79	36.84	7.75	0.22
Coefficient of variation (%)	4.77	20.86	16.21	39.23	20.46	19.53	44.47	26.77	57.34	32.83

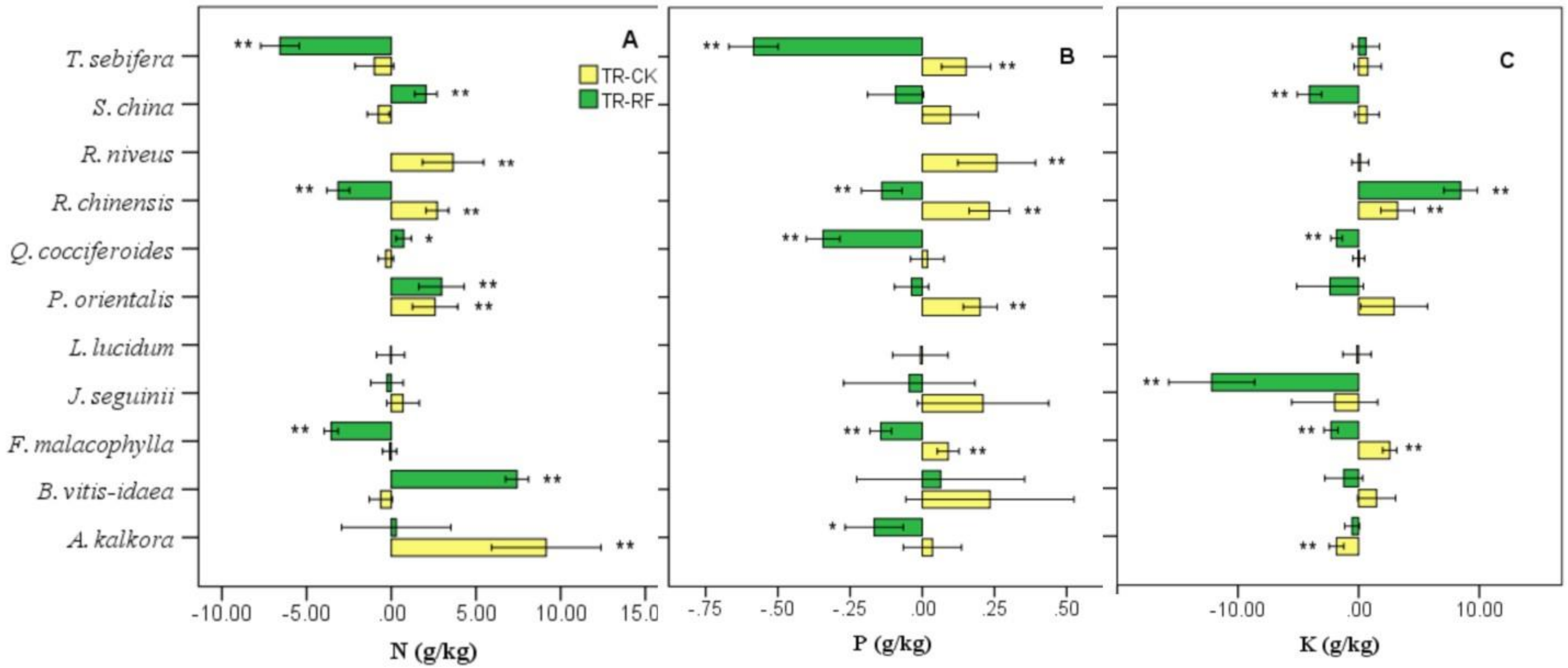
A
B
C
TR-CK
TR-RF
T. sebifera
S. china
R. niveus
R. chinensis
Q. cocciferoides
P. orientalis
L. lucidum
J. seguinii
F. malacophylla
B. vitis-idaea
A. kalkora
-10.00
-5.00
.00
5.00
10.00
15.0
N (g/kg)
-.75
-.50
-.25
.00
.25
.50
P (g/kg)
-10.00
.00
10.00
K (g/kg)

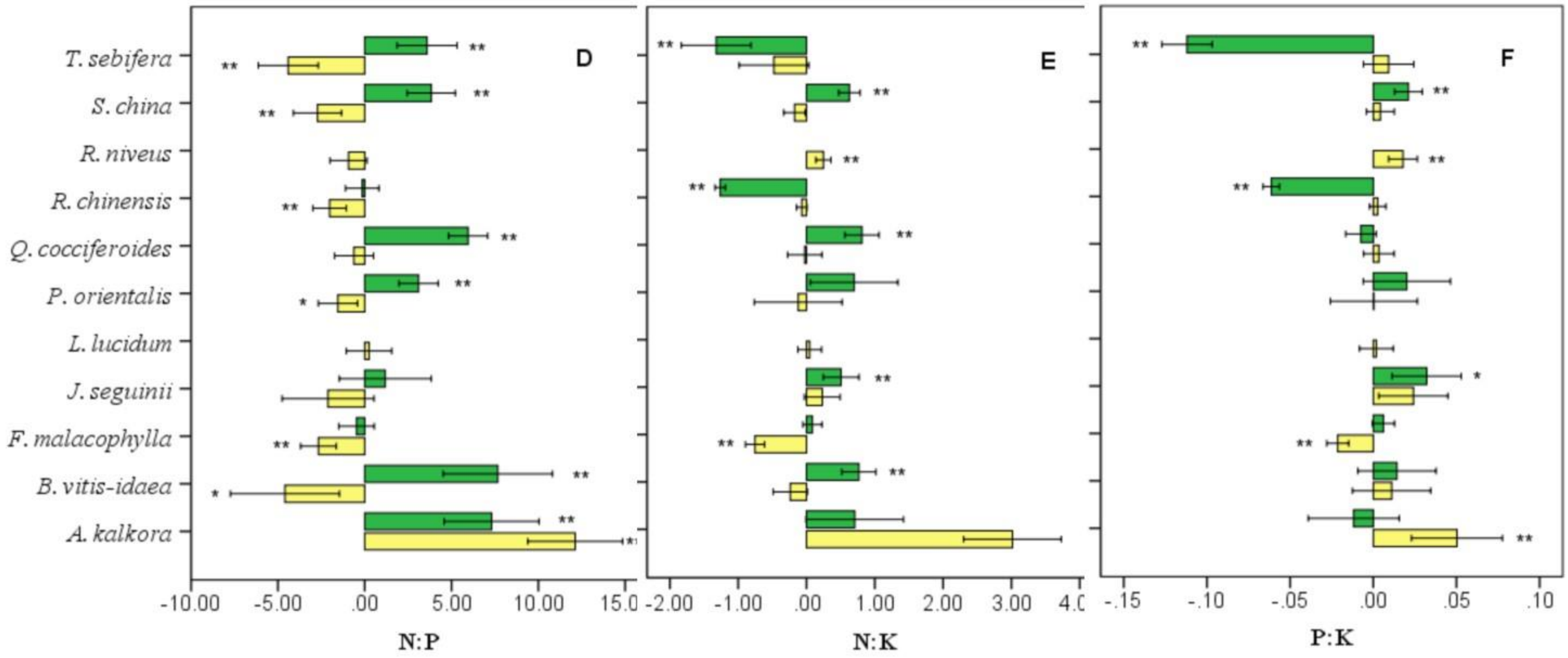

Figure S1 Comparison of elemental concentrations (A–C) and ratios (D–F) of the same species growing in managed treatments (TR, shading and/or watering, eight in total) with the counterparts in the control treatment (CK quadrats) and a natural reference forest. The significance of the ratio difference for the corresponding species was tested using a one-sample t-test. The yellow bars are results of the managed treatments vs. controls; green bars are results of the managed treatments vs. natural forest. * is significant at 0.05, ** is significant at 0.01, and no symbol implies no significance. The *Ligustrum lucidum* and *Rubus niveus* lack of seedlings in the forest for the comparison.

Table S2 Stoichiometric indices of leaf were calculated at the community level in three ways

Type of community mean	Formula	Note
(a) Arithmetic mean	$EC_{com,1} = \frac{\sum C_i}{N}$	where $EC_{com,2}$ is the elemental concentration of C, N, P or K at the community level; C_i is the concentration for a given species (n = 11); N is the number of study species (n = 11).
(b) Mean weighted by abundance	$EC_{com,2} = \frac{\sum (C_i \times N_i)}{\sum N_i}$	where $EC_{com,2}$ is the elemental concentration of C, N, P or K weighted by species abundances at the community level; C_i is the concentration for a given species (n = 11); N_i is the number of individuals of a given species (n = 11).
(c) Mean weighted by leaf biomass	$EC_{com,3} = \frac{\sum (C_i \times M_i)}{\sum M_i}$	where $EC_{com,3}$ is the elemental concentration of C, N, P or K weighted by leaf biomass at the community level; C_i is the concentration for a given species (n = 11); M_i is the dry mass of leaves for a given species (n = 11).
	$M_i = m_i \times n_i \times N_i$	where m_i is the mean dry mass of a single leaf of given species; n_i is the mean number of leaves per individual; N_i is the number of individuals of given species in a particular treatment.
